# Poor performance of open incisional biopsy for the microbiological diagnosis of periprosthetic knee joint infection

**DOI:** 10.1038/s41598-021-90475-1

**Published:** 2021-05-26

**Authors:** Jan Schwarze, Burkhard Moellenbeck, Georg Gosheger, Tom Schmidt-Braekling, Lukas Lampe, Sebastian Klingebiel, Thomas Ackmann, Christoph Theil

**Affiliations:** Department of Orthopedics and Tumor Orthopedics, Muenster University Hospital, Muenster, Albert-Schweitzer-Campus 1, 48149 Muenster, Germany

**Keywords:** Outcomes research, Medical research

## Abstract

The accurate preoperative diagnosis of periprosthetic joint infection (PJI) of total knee arthroplasty (TKA) can be difficult despite the use of a combination of serum and synovial markers. In such inconclusive cases, incisional open biopsy might be considered. This study investigates the usefulness of biopsies in patients with inconclusive diagnostic findings. We retrospectively identified 63 patients who underwent incisional biopsy for chronic PJI in the operation theatre following TKA revision between 2010 and 2018 after inconclusive preoperative diagnostics for PJI. In all cases, 5 independent biopsies were taken. Results from open biopsy for PJI were analyzed for diagnostic accuracy using the intraoperative results from following revision surgery as gold standard. 27 patients (43%) had a positive culture taken during biopsy. 15 cases (24%) met the diagnostic criteria for a chronic PJI. Most common organisms were Coagulase*-*negative staphylococci (67%) and *Cutibacterium acnes* (30%). Compared to the findings during revision surgery, biopsies showed a sensitivity of 47% and a specificity of 77% for PJI. Open incisional biopsy following inconclusive serum- and synovial diagnostics for low grade PJI may be considered for identification of microorganisms. Due to its low sensitivity and moderate specificity found in the present cohort, microbiological analysis should be combined with additional diagnostic markers and histological investigation.

Level of Evidence. Retrospective cohort study (Level III).

## Introduction

Periprosthetic joint infection (PJI) following total knee arthroplasty (TKA) is a rare but serious complication^1,2^ that is a leading cause for revision and can be associated with devastating consequences including high mortality of up to 21% after 5 years and repeat revision surgeries^3–5^. An accurate diagnosis or exclusion of PJI is paramount prior to revision surgery, but can be very difficult in low-grade infections in which common clinical signs are often absent^1,6^. The high prevalence of pain following primary and revision TKA further complicates clinical diagnosis and surgeons must rely on a combination of serum and synovial markers and microbiological findings to diagnose infection^7^. Currently, the Musculoskeletal Infection Society (MSIS) or International Consensus Meeting (ICM) criteria that incorporate these diagnostic tools in a scoring system are widely used^8,9^. However, while the sensitivity and specificity of individual tests and a combination of tests is high, there are challenging cases that don’t fulfil the criteria for infection, but present with a high suspicion for PJI^8^. Despite the high sensitivity and specificity of synovial markers there are cases of a dry tap, clotted specimens or blood contamination of the aspirate, which have been described in up to 36% that greatly impair the quality of synovial analysis^10,11^. Moreover even if all necessary diagnostic parameters according to the MSIS Criteria have been analyzed an inconclusive score (“possibly infected”) (2–5 points) may occur and further investigation is recommended in these cases^8^. Furthermore, microbiological cultures from synovial fluid vary greatly in their accuracy in the identification of the causative pathogen in PJI depending on the underlying study. A range of sensitivities from 12 to 94% has been reported by Patridge et al.^12^, complicating targeted antibiotic treatment.

A potential option for patients in whom PJI is suspected, but preoperative diagnostic workup has been inconclusive, joint biopsy with sampling of periprosthetic and synovial tissue has been proposed. These procedures can be performed arthroscopically, through needle biopsy or as incisional open biopsies with no approach being clearly superior to the others^13–16^. Previous studies reported high sensitivity and specificity for routine synovial biopsies in the diagnosis of low-grade chronic PJI^13,14,17–19^. However, there is a lack of studies on open synovial biopsies in cases of inconclusive or intermediate preoperative score results in the diagnostics for chronic PJI in TKA. This study investigated if there is a benefit of open incisional biopsies after inconclusive results of preoperative serum and synovial markers.

## Methods

Ethical approval was obtained by the local ethics committee (Ethik Kommission der Ärztekammer Westfalen-Lippe und der Westfälischen Wilhelms-Universität ref. no 2019-728-f-S). The study was conducted according to the principles of the declaration of Helsinki by the World Medical Association.

In a retrospective analysis of revision arthroplasty database (2010–2018), we identified 63 patients with a painful or radiographically loosened TKA (Table 1), that underwent an open incisional biopsy for further evaluation after inconclusive prior diagnostics for PJI. A biopsy was offered prior to revision surgery if patients either had an intermediate ICM score (2–5 points) or had a dry tap or clotted aspirate. Sixteen patients without following revision surgery in our institute were excluded.

**Table 1:** Patients’ details.

Variable	N (total 63)	%*
Male sex	41	65
Type of TKA*		
Primary	21	33
Revision implant^a^	41	65
Megaprosthesis	1	2
**Radiographic imaging**		
No Signs for loosening	32	51
Signs of loosening	24	38
Osteolysis	7	11
**Previous revision surgery**		
0	11	18
1	13	21
2	17	27
3	6	10
4 or more	16	25
Previous PJI	23	37

^a^constrained TKA.

*Total percentage may be > 100% or < 100% due to rounding.

All patients underwent analysis of serum inflammatory markers (c-reactive protein (CRP), serum white blood cell count (WBC) and serum interleukin-6 (IL-6)) and a sterile joint aspiration. Synovial fluid leukocyte count as well as percentage of neutrophils was determined, and fluid samples were sent for microbiological analysis (Table 2). Since only TKAs were included in this study a leukocyte count ≥ 1700/microliter or percentage of neutrophils ≥ 65% in the fluid leukocyte differentiation was considered suspicious for PJI^20^.

**Table 2:** Preoperative diagnostics.

Variable	N	%*	
**Serum markers**			
CRP ≥ 10 mg/l	28/63		44
IL-6 ≥ 3 pg/ml	18/24		75
**Joint aspiration**			
Dry tap	22/63		35
Clotted aspirate	6/63		10
Leucocytes count ≥ 1700/µl	19/40		48
PMN^1^ ≥ 65%	19/39		49
Positive culture	3/44		7

**Figure 1:**
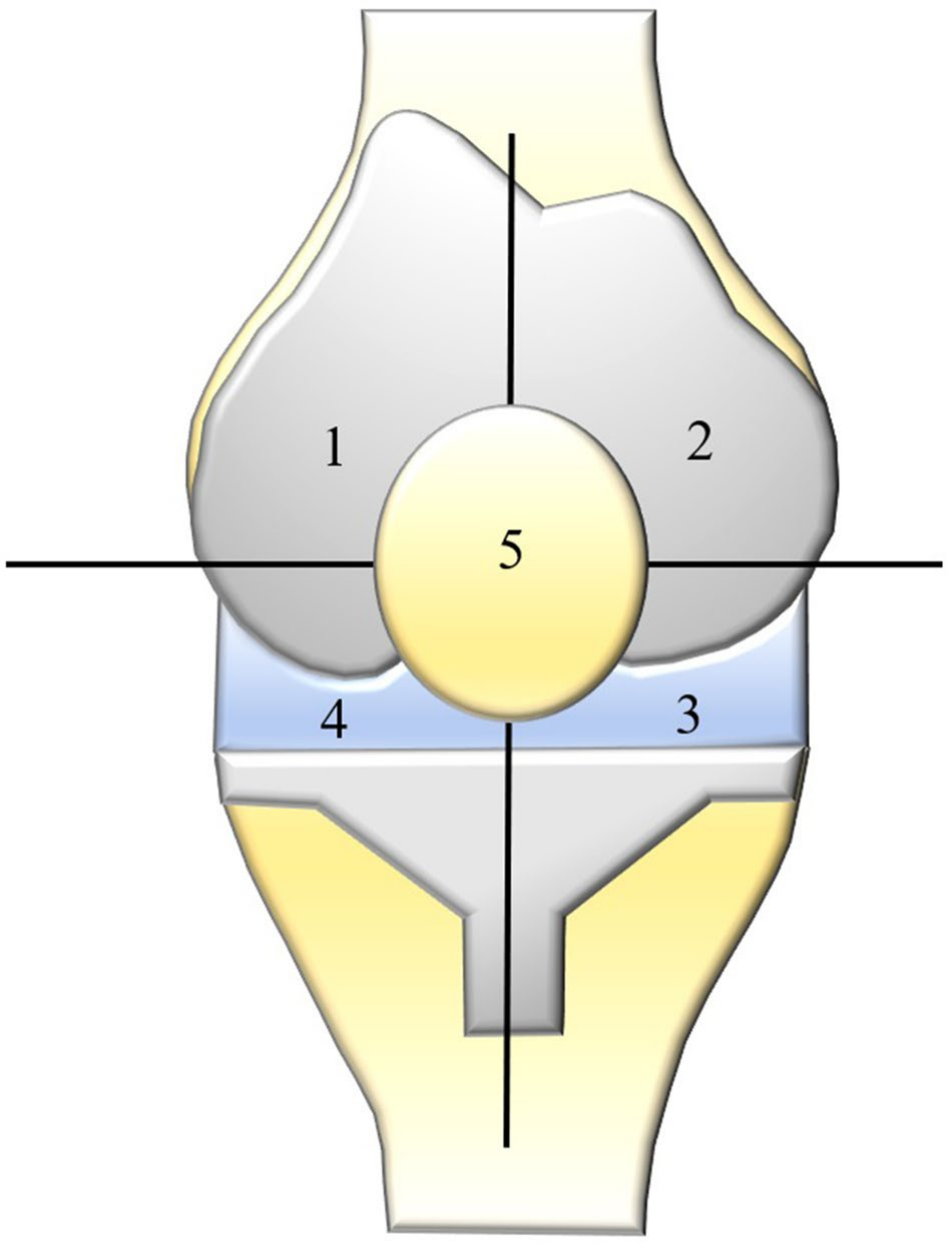
Schematic visualization of open incisional biopsy. Tissue was obtained individually superior to the patella medial (1) and lateral (2), inferior to the patella medial (3) and lateral (4) as well as from the patella (5).

Conventional radiographs were checked for signs of implant loosening or osteolysis. Implant loosening was suspected when radiolucent lines in a.-p. and lateral radiographs amounted to ≥ 2 mm applying the criteria by the Knee Society Scoring System^21^. After patients’ informed consent all open biopsies were performed under strictly sterile conditions in the operation theatre. After standard disinfection using alcohol disinfectant (Codan Schülke & Mayer, Zürich, Switzerland) for a minimum of five minutes and sterile draping an iodine-impregnated incision drape (3 M™ Ioban™ antimicrobial incision drape) was generally applied. No tourniquet was used. Though a suprapatellar mini-arthrotomy of 3-5 cm a minimum of 5 synovial biopsies for microbiological analysis were taken. The open biopsies were generally taken in a strictly standardized manner to gain a most representative result: tissue was obtained individually superior to the patella medial and lateral, inferior to the patella medial and lateral as well as from the patella (Fig. 1).

For every biopsy site, a new forceps was used to prevent contamination. Following the sampling a perioperative intravenous antibiotic prophylaxis with cefuroxime 1.5 g was applied (or clindamycin 600 mg in cases of penicillin allergy) and irrigation with sterile Ringers lactate solution was performed. Each sample was preserved in a sterile tube and immediately sent for culturing. All samples were cultured on chocolate agar, Schaedler agar and Columbia agar for a period of 10–14 days. If a fungal infection was suspected, chromogenic agar and Kimmig agar was used in addition^22^. All cultures were checked daily for growth. They were declared negative if no growth was detected within 10–14 days of cultivation. Microorganisms were analyzed using standard microbiological procedures for bacterial differentiation and antibiotic resistant patterns.

On basis of the microbiological findings and preoperative score for PJI TKAs were classified as aseptic or PJI according to the ICM Criteria. During revision surgery following the biopsy, a minimum of three to five intraoperative deep tissue samples for additional microbiological analysis were taken and processed as described above. The culture results of incisional biopsies were compared to the culture results of revision surgery. To calculate sensitivity and specificity of incisional biopsy, diagnosis of PJI based on ICM criteria with respect to the findings of revision surgery were used as gold standard.

### Statistical analysis

Patients’ data were anonymized prior to analysis. Statistical analysis was performed using SPSS Statistics for Windows Version 25 (IBM Corporation, Armonk, NY, USA). Depending on the distribution of data, medians and 25–75% interquartile ranges (IQR) for non-parametric data and means and ranges for parametric data are presented. Sensitivity and specificity of the open incisional biopsy was calculated using the microbiological findings of patients that received revision surgery of the TKA of any kind. Differences between groups were analyzed via crosstables and the chi-squared-test for categorical variables, while metric data were compared using the Mann–Whitney U-test or students’ t-test depending on the distribution of data. Possible risk factors for positive cultures in the open biopsy or in the revision surgery underwent a univariate analysis using the Cramer-V test for categorical variables.

## Results

Microbiological findings from open biopsy showed positive cultures in 27 patients (43%). Based on these findings 19 patients (30%) were diagnosed with PJI.

The most frequent microbiological findings included (CONS) in 18 cases (67%) and *Cutibacterium acnes species* (ssp.) in 8 of cases (30%). Polymicrobial findings (≥ 2 different species) were present in 7 cases (26%) (Table 3).

**Table 3:** Pathogens.

Positive cultures	N (total 27)	%
CONS	18	67
*Cutibacerium* acnes	8	30
*Enterococcus* sp.	1	4
*Candida* ssp.	1	4
Polymicrobial^a^	7	26

^a^ ≥ 2 different pathogens, Total number/percentage of positive cultures is > 27 / > 100% because of polymicrobial findings in 7 cases.

With the numbers available, serum CRP ≥ 10 mg/l, , previous history of PJI, elevation of leukocyte count ≥ 1700 µl, or PMN ≥ 65% in the joint aspirate were not correlated with the diagnosis of PJIin the open biopsy (p = 0.16; p = 0.31; p = 0.94; p = 0.84 Cramer V test).

One patient developed a wound healing disorder after open biopsy and resulted in a PJI and subsequent two-stage exchange of the TKA. Further complications could not be observed. The most frequent pathogens at the time of revision were CONS (55%) and *Cutibacterium acnes (14%)* or *Enterococcus species* (14%). In contrast to the findings in the open biopsy highly virulent pathogens like *methicillin sensitive Staphylococcus aureus (MSSA)* and *Klebsiella pneumoniae* were also found (Table 4).

**Table 4:** Pathogens in revision surgery.

Positive cultures	N (total 29)	%
Total	29	100
CONS	16	55
*Cutibacterium* acnes	4	14
*Enterococcus* sp.	4	14
*Bacillus cereus*	3	10
*MSSA*	2	7
*Candida* ssp.	1	3
*Streptococcus* ssp.	1	3
*Klebsiella pneumoniae*	1	3
*Dermabacter hominis*	1	3
Polymicrobiological^a^	4	14

^a^ ≥ 2 different pathogens, Total number/percentage of positive cultures is > 29/> 100% because of polymicrobial findings.

The median time to revision was 1.8 month (IQR 1.3–2.9 month). In patients that were diagnosed with PJI at the time of open biopsy (n = 19), the diagnosis of PJI was confirmed in 9 cases using the intraoperative samples taken during revision surgery as gold standard.

In contrast, among the 44 patients that were considered to have aseptic failure and underwent single stage revision, unexpected positive cultures were found in 23% (10/44 patients) changing the diagnosis to PJI. On the other hand, in 67% (34/44 patients) the diagnosis of aseptic failure was confirmed.

The calculated sensitivity of open incisional biopsies amounted to 47%, a positive predictive value of 39%, a specificity of 77% and a negative predictive value of 62% for chronic PJI (p = 0.05 Chi Square Test) (Table 5).

**Table 5:** PJI in revision surgery vs. diagnosis of PJI—open biopsy.

		PJI—open biopsy		Total
		No	Yes	
PJI in revision surgery	No	34	10	44
	Yes	10	9	19
Total		44	19	63

## Discussion

The correct diagnosis of low-grade PJI in TKA following either dry tap joint aspiration or uncertain results in the preoperative diagnostic workup remains one of the most difficult challenges for the orthopedic surgeon. An undetected low grade infection prior to a partial or complete exchange of the prosthesis may result in repeated revisions, prolonged hospitalization and increased morbidity and mortality^23,24^. While Microbiological cultures are a useful tool in the detection of a PJI and offer the opportunity to establish a tailored antibiotic treatment, reported incidences of culture negative PJI from 7 to 42% show the urgent need for further improvement of isolation and identification of the respective organism^25^.

Several studies reported high sensitivity and specificity of routine biopsies in the diagnosis of PJI^14,16,17,19^. In a prospective evaluation of joint aspiration and biopsy by Meermans et al. using a needle technique in 120 patients with suspected PJI, aspiration showed a sensitivity of 83% and 79% for the biopsy with 100% specificity for both approaches^16^. In a recent study, Fink et al. presented comparably accurate results using an arthroscopically guided biopsy forceps in routine biopsies (sensitivity 93.8% specificity 97.3%) in cases of implant loosening in a large cohort of 508 patients (277 TKA, 237 THA)^19^, which emphasized the positive results from the same group assessing unguided synovial biopsies performed for the same indication in 145 TKAs (sensitivity 100%, specificity 98.1%)^14^. These results lead to the conclusion that a routinely performed biopsy without prior selection of cases with an inconclusive prior diagnostic work-up for PJI in TKA before revision surgery is a reliable diagnostic tool regardless of the chosen device.

In contrast to these findings, a prospective comparison of guided vs. unguided biopsy for histological and microbiological diagnostics in 40 patients with chronic pain after TKA and inconclusive joint aspiration, Scheele et al. reported negative results for PJI in 36 cases with intraoperative positive findings in the following revision in 4 patients. Unfortunately, all four patients with a suspicious biopsy for PJI denied a revision of the TKA making determination of sensibility and specificity impossible^13^. Nevertheless, this data already alludes to the challenge of organisms isolation and correct diagnostic of low grade PJI following previous inconclusive diagnostics. To our knowledge, the present study is the first to assess specificity and sensitivity of open incisional biopsies following an inconclusive diagnostic workup and comparison with intraoperative tissue samples from the subsequent TKA revision.

The open incisional biopsy technique for microbiological investigation alone shows a poor performance in this particular cohort with a sensitivity of only 47% and a specificity of 77% for a positive result in following revision surgery. As the indication for open biopsy in the present study was inconclusive prior diagnostic work-up and we did not routinely perform synovial biopsies prior to a planned revision of a TKA, our results could be influenced by the fact that patients with conclusive serum and synovial markers that already either ruled out or confirmed a PJI where not included in our cohort.

The findings of this study must be interpreted considering several limiting factors. Due to its retrospective design and small collective of possible low-grade PJI, there might be potential factors that impact these findings that were not accessible for analysis. In addition the underlying gold standard of this study may be impeded by the fact that the amount of perioperative tissue samples differs from three to five samples. Furthermore, while these results were collected over a period of nine years during which the MSIS and ICM criteria for PJI have evolved over time and novel markers and criteria have been added more recently that were not analyzed in the present cohort. Moreoverwhile serum CRP analysis was performed for every case, obligatory analysis of serum IL-6 was later added to our institution’s diagnostic algorithm for PJI and therefore only available in 34 cases (Table 2). Because of its high sensitivity for infection IL-6 could be a useful addition in the diagnostic of low-grade PJI and should further be evaluated^26,27^. The lack of routinely performed histological analysis of the gained tissue samles presents a further limitation From 2010–2018 it only performed if an osteolysis was visible on the preoperative radiographs for additional investigation for malignancy.

In our institution’s current practice, whenever open biopsy is recommended to confirm or exclude low-grade PJI we conduct collection of microbiology tissue samples as described, as well as histological sampling, a quantitative alpha-defensin analysis and additional cell count and differential by an outside laboratory^28^. For every revision arthroplasty with complete or partial exchange of the prosthesis sonication is used in our department in addition to synovial aspiration and tissue sampling^29^.

## Conclusion

Open incisional biopsy for microbiological analysis in cases of prior inconclusive serum- and synovial diagnostics for low grade PJI may be considered for isolation of possible microorganisms. If PJI is ruled out by open biopsy, an aseptic approach for revision surgery is reasonable. However, due to its low sensitivity and moderate specificity found in the present cohort, an invasive procedure must be weighed carefully, and microbiological analysis should be combined with additional diagnostic markers and histological investigation.

## Data Availability

The datasets generated during and/or analysed during the current study are not publicly available due to our institutions regulations of data privacy but are available from the corresponding author on reasonable request.

## Abbreviations

CDC: Centre of disease control
CONS: Coagulase-negative staphylococci
CRP: C-reactive protein
ICM: International consensus meeting
IDSA: Infectious diseases Society of America
IL-6: Interleukin-6
MSIS: Musculoskeletal Infection Society
OR: Odd ratio
PJI: Periprosthetic joint infection
TKA: Total knee arthroplasty
WBC: White blood cell count
